# When digital-AI transformation sparks adaptation: job crafting and AI knowledge in job insecurity contexts

**DOI:** 10.3389/fpsyg.2025.1612245

**Published:** 2025-08-14

**Authors:** Chengcheng Sha, Tianlong Chai

**Affiliations:** ^1^Department of Economics and Trade, Guangxi Eco-Engineering Vocational & Technical College, Liuzhou, China; ^2^School of Civil Engineering and Architecture, Guangxi University of Science and Technology, Liuzhou, China

**Keywords:** digital-AI transformation, job insecurity, job crafting, AI knowledge, conservation of resources theory

## Abstract

**Introduction:**

As AI technology continues to rise, numerous studies have explored its impact on employee behavior. However, little is known about employees’ responses to the integration of AI in the digital transformation process. Drawing on Conservation of Resources Theory, this study aims to examine the impact of digital-AI transformation on employees’ job crafting behaviors, focusing on the mediating role of job insecurity and the moderating effect of AI knowledge.

**Methods:**

A two-wave survey was conducted among 400 employees actively using AI tools in digitally transforming organizations, resulting in 370 valid responses. Data were analyzed using SPSS 22.0 and the PROCESS macro (version 3.3) to test the proposed hypotheses.

**Results:**

The results indicate that digital-AI transformation has a significant positive effect on employees’ job crafting (*β* = 0.512, *p* < 0.001), with job insecurity serving as a mediator in this relationship (*β* = 0.228, *p* < 0.001). Employees’ AI knowledge not only moderates the positive effect of digital-AI transformation on job crafting (*β* = 0.060, *p* < 0.05), but also moderates the mediating role of job insecurity in the relationship between digital-AI transformation and job crafting (*β* = 0.143, *p* < 0.001).

**Discussion:**

This study extends the application of Conservation of Resources Theory by emphasizing the potentially positive role of job insecurity under specific contextual conditions, while also offering a critical reflection on the ethical implications of using job insecurity as a motivational tool. It is suggested that organizations should leverage employees’ AI knowledge to enhance job crafting, rather than relying on stress as a driver. Future research is encouraged to explore additional antecedents of job crafting.

## Introduction

As artificial intelligence (AI) technology continues to rise, it has become a primary driver of digital transformation across industries (Kaplan and Haenlein, 2019; Wu et al., 2024). According to a 2023 survey conducted by the large U.S.-based job site Resume Builder (2023), 49% of companies report using ChatGPT, with 93% indicating plans to expand their use of chatbots. ChatGPT is applied in various internal functions, such as recruitment and coding. These shifts reflect the growing influence of AI in reshaping how organizations operate and how employees work. While AI can enhance productivity and streamline organizational processes (Jarrahi, 2018), it also poses significant challenges for employees, particularly in the human resource domain (Strohmeier, 2020). This AI-driven digital transformation is therefore seen as a double-edged sword—offering efficiency gains on the one hand, and psychological and occupational uncertainty on the other.

Despite increasing attention to the organizational implications of AI (Duan et al., 2019; Ho et al., 2022), existing studies have primarily focused on its negative impacts on employees, such as stress and anxiety (Stich et al., 2019; Wang and Wang, 2022). Less is known about how employees actively respond to such transformations, and under what conditions they may play constructive roles. This is a critical gap, as employees are not merely passive recipients of technological change. They can also adapt, reshape, and even co-create their roles through behaviors such as job crafting (Zhao et al., 2025). Job crafting enables employees to proactively redesign their tasks, relationships, and work perceptions, which has been shown to support technological change and enhance individual performance (Fenwick et al., 2024; Teng, 2019).

In this study, we argue that AI knowledge plays a foundational role in how employees navigate digital-AI transformation. AI knowledge—defined as employees’ familiarity and comfort with AI technologies (Chiu et al., 2021), can shape their capacity to cope with AI-related demands. According to Conservation of Resources Theory, individuals experience stress when they anticipate the loss of personal resources (Bickerton and Miner, 2023; Hobfoll, 1989). Employees encountering AI-driven changes may perceive threats to their skills, relevance, or job security, especially given the complexity of new AI tools (Wang et al., 2022). This perceived job insecurity may undermine their proactive engagement. However, Conservation of Resources Theory also suggests that personal resources like AI knowledge can buffer against such negative effects and promote adaptive behavior (Bakker and Demerouti, 2017; He et al., 2024).

Current research on AI-driven technological change primarily focuses on low-skill industries, as these occupations are more susceptible to replacement by AI, potentially triggering emotional and behavioral responses among employees (Rampersad, 2020). For instance, such effects have been observed in sectors such as e-commerce, hospitality, and manufacturing (Cheng et al., 2023; He et al., 2024; Sha et al., 2025). The impact of AI, however, varies across industries. In sectors like technology and finance, AI tools are typically employed to enhance decision-making and improve efficiency, thereby empowering employees to redefine their roles (Manser Payne et al., 2021). In contrast, in industries such as manufacturing and logistics, AI is often perceived as a substitute for routine tasks, which may exacerbate perceptions of job insecurity (Ojiyi et al., 2023). These sectoral differences are likely to influence how employees perceive threats and engage in job crafting. Therefore, a cross-industry sample is adopted in this study to provide a more comprehensive understanding of employees’ psychological and behavioral responses to digital-AI transformation and to enhance the generalizability of the findings.

Based on the analysis above and drawing from Conservation of Resources Theory, we constructed a moderated mediation model to investigate how employees play an active role during digital-AI transformation and to uncover its underlying mechanisms. This study addresses three primary research aims:

1. To examine how digital-AI transformation influences employees’ job crafting;
2. To test the mediating role of job insecurity within the proposed model;
3. To explore how AI knowledge, as a personal resource, moderates the impact of digital-AI transformation.

This study has the following contributions: first, it expands research on the impact of AI-driven digital transformation within human resource management. Second, by introducing job insecurity as a variable, it explores the motivations for employees’ proactive engagement in digital-AI transformation, addressing a gap in research on the positive implications of AI adoption. Finally, we integrate AI knowledge as a personal resource within the Conservation of Resources framework to explore effective strategies that foster proactive roles among employees in the context of digital-AI transformation.

## Theoretical overview and hypotheses

### Digital-AI transformation and job crafting

Employees often adopt a conservative stance toward organizational technological change, primarily due to perceived uncertainties associated with such transitions (Arias-Pérez and Vélez-Jaramillo, 2022). However, driven by the wave of digital transformation, many companies are actively embracing AI to reshape their business models (Butner and Ho, 2019). The emergence and widespread application of new technologies are sparking profound technological changes, thereby intensifying employees’ concerns over job insecurity and workplace competition (Oztemel and Gursev, 2020; Petrick, 2023). Conservation of Resources Theory posits that individuals tend to protect and acquire resources when facing stress, threats, or potential losses, aiming to prevent further resource depletion (Kuzgun et al., 2023). Under the pressures and challenges of digital-AI transformation, employees invest resources to minimize resource loss and mitigate perceived threats (Bickerton and Miner, 2023). Specifically, when employees face pressure resulting from technological change, the stressor is often perceived as a challenge at work, prompting them to take adaptive actions (Webster et al., 2011). They may adopt strategies to adapt to uncertainties brought by digital-AI transformation, actively acquiring new knowledge and skills to meet the demands of this transition (Liang et al., 2022; Uzule and Verina, 2023). Moreover, digital-AI transformation provides opportunities for resource acquisition, fostering employees’ innovative mindset and motivation, which encourages proactive changes in their work approaches (Furjan et al., 2020; Meijerink et al., 2020). Not only that, when employees are confronted with digital-AI transformation within their organization, the integration of AI is often perceived as a threat to job security (Sha et al., 2025). As a result, the transformation tends to be viewed as a work-related threat, leading employees to engage in defensive job crafting behaviors (Cheng et al., 2023).

In the process of digital-AI transformation, the integration of AI technology grants employees greater autonomy and flexibility in their roles (Nambisan et al., 2019). To safeguard their resources and ensure ongoing growth within the organization, employees seek avenues for self-transformation beyond fulfilling their standard duties (Tan et al., 2024). Concurrently, digital-AI transformation brings new technology integration and business model innovation, which may render existing skills obsolete. To maintain competitive advantage, employees must demonstrate adaptability, learning capacity, and willingness to embrace change (Wang et al., 2022). Job crafting, defined as the proactive adjustments employees make to their tasks and perceptions to find renewed work meaning and meet organizational demands, serves as a way to reshape work identity (Tims et al., 2022). Previous empirical studies have shown that organizational AI-driven technological change tends to activate employees’ job crafting behaviors (An and Hu, 2025; Cheng et al., 2023; Xiao et al., 2025; Wang et al., 2025). Building on this evidence, it is hypothesized that, when facing digital-AI transformation, employees are more likely to proactively initiate changes to protect their personal resources.

*H1*: Digital-AI transformation in enterprises positively influences employee job crafting.

### The mediating role of job insecurity

Job insecurity has varied definitions depending on the context, but it is commonly understood as an individual’s perception of employment stability within an organization (Reisel et al., 2010). In the context of digital-AI transformation, job insecurity refers to the perceived risk of job displacement due to AI adoption and the sense of inadequacy arising from the need for continuous skill updates for effective human-machine collaboration (Wu et al., 2024). This perception embodies employees’ concerns about their future career prospects and their awareness of factors within the work environment that may threaten their growth potential (Kim and Beehr, 2023; Yi et al., 2022). Digital transformation reshapes business processes within organizations, introducing technological changes and structural adjustments that heighten internal uncertainty and employees’ job insecurity (Brett and Drasgow, 2002; He et al., 2024). Routine tasks increasingly require less human intervention, rendering some roles redundant and causing employees to perceive threats to their resources, leading to concerns about current job stability and future career prospects (Li et al., 2019). Simultaneously, employees face the need to acquire new skills and tools in response to the challenges of digital-AI transformation; however, mastering these skills in a short time proves challenging, often resulting in elevated psychological stress, frustration, and job insecurity (Rangrez et al., 2022; Wang and Wang, 2022).

While digital-AI transformation may lead to job insecurity and other adverse effects, causing employees to feel threatened and stressed, it does not necessarily result in negative outcomes (Tan et al., 2024). On the contrary, the pressure associated with AI advancements can motivate employees to proactively seek strategies to adapt within their organizations (Liang et al., 2022). According to the Conservation of Resources Theory, when individuals perceive a threat of resource loss, their stress levels increase accordingly (Bickerton and Miner, 2023). Under such pressure, individuals tend to seek new resources and actively adjust their behavior to align with organizational goals (Hobfoll et al., 2018). Moreover, when employees experience job insecurity, it signifies their recognition of resource threats (Sverke et al., 2002). Consequently, to safeguard their resources, employees may consciously invest additional time and effort into learning new skills and knowledge, aiming to enhance work processes and organizational performance, thereby increasing their value within the organization (Sun et al., 2022; Tannenbaum and Wolfson, 2022). Additionally, employees facing job insecurity are likely to take proactive steps to adapt to a rapidly changing work environment, initiating actions to improve their circumstances (Meijerink et al., 2020). In summary, job insecurity may motivate employees to protect their resources, encouraging them to acquire new skills and refine current work practices. We propose the following hypothesis:

*H2*: Job insecurity mediates the relationship between digital-AI transformation and job crafting among employees.

### The moderating and moderated mediation effects of AI knowledge

AI knowledge is defined as an individual’s subjective perception of AI, rather than an objective assessment of actual expertise (Chiu et al., 2021; He et al., 2024). A related concept is AI literacy, which refers to an individual’s ability to critically engage with AI technologies and reflects a deeper understanding of AI (Bewersdorff et al., 2025). In contrast, AI knowledge primarily captures users’ superficial or general awareness of AI. During digital-AI transformation, employees’ superficial understanding of AI may serve as an amplifying factor.

According to Conservation of Resources Theory, individuals display a strong motivation to protect their resources. When they perceive a potential loss, they tend to utilize their existing resources to prevent further depletion (Meijerink et al., 2020). This reflects individuals tendency to invest their remaining resources strategically in order to prevent further depletion and regain control over their work environment. AI knowledge reflects employees’ understanding of AI technologies and their development, serving as a critical personal resource during digital-AI transformation (Chiu et al., 2021; He et al., 2024). Specifically, AI knowledge, as a form of personal resource, exerts both buffering and resource-amplifying effects. For instance, when employees experience role uncertainty due to digital-AI transformation, those with higher AI knowledge are more likely to interpret these changes as opportunities for growth rather than threats to employment, thereby buffering the negative impact (Chiu et al., 2021). Moreover, employees with greater familiarity with AI actively leverage their expertise to seek training opportunities and explore ways to integrate AI into their work practices (Hu et al., 2025; Tan et al., 2024). In contrast, employees with limited AI knowledge tend to feel overwhelmed and fail to perceive digital-AI transformation as an opportunity, which may reduce their motivation for job crafting. Thus, AI knowledge not only functions as a buffer that alleviates job insecurity during AI-driven change but also serves as a resource amplifier that enhances employees’ confidence in engaging in job crafting throughout the transformation process.

Firstly, we propose that AI knowledge can amplify the positive effects experienced by employees during the digital-AI transformation. High levels of AI knowledge enable employees to focus more on the beneficial aspects of AI technologies (He et al., 2024). According to resource conservation theory, job insecurity induces stress in employees, leading to the depletion of their resources (Bickerton and Miner, 2023). When employees perceive a loss of resources, they strive to replenish those losses. As organizations begin to integrate AI technologies into their digital-AI transformation processes, employees with higher AI knowledge are more likely to actively invest personal resources to reshape their work practices and adapt to the technological changes within the organization (He et al., 2024). Therefore, AI knowledge can strengthen the impact of digital-AI transformation on employees’ job crafting.

Furthermore, we propose that AI knowledge can moderate the impact of digital-AI transformation on job crafting through job insecurity. Despite the rapid advancement and powerful capabilities of AI technologies, they still exhibit certain limitations. A study on employees’ attitudes towards the application of AI technologies in organizations indicates that most employees hold a positive outlook and believe they will not be replaced by AI (Oh et al., 2019). Specifically, employees with a strong understanding of AI knowledge recognize the limitations and current development levels of AI, acknowledging that AI cannot fully replace humans in the short term (He et al., 2024). Therefore, during the process of digital-AI transformation, employees with high levels of AI knowledge are likely to mitigate the impact of job insecurity. They perceive the integration of AI technologies as an opportunity within the organizational digital transformation journey and actively pursue self-transformation to adapt to the digital-AI transition. Based on the above analysis, we propose the following hypotheses:

*H3*: AI knowledge moderates the relationship between digital-AI transformation and Job crafting.

*H4*: AI knowledge moderates the mediating effect of job insecurity on the relationship between digital-AI transformation and Job crafting.

**Figure 1:**
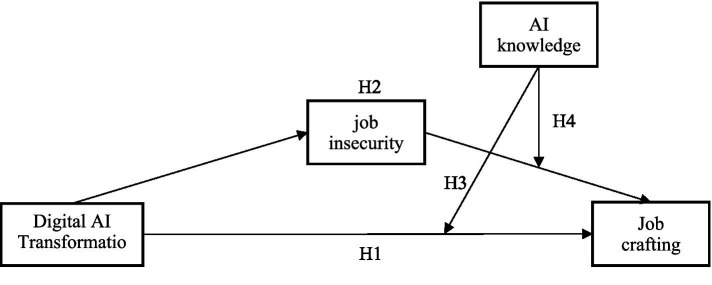
Theoretical model of the study.

To explore the impact mechanism of digital-AI transformation on Job crafting, a theoretical model was developed, as illustrated in Figure 1.

## Method

### Sample and procedure

In this study, we collected data through an online survey distributed via Credamo, a widely-used survey platform in China, favored by many Chinese scholars (Liang et al., 2022; Wu et al., 2024). Participants were explicitly informed of the survey’s purpose before their involvement, ensuring that all collected information would be used solely for this study. To ensure that respondents were associated with companies undergoing digital-AI transformation, we provided a detailed explanation of digital-AI transformation at the beginning of the questionnaire. Additionally, we included a screening question, “Is your company currently undergoing digital-AI transformation?” to verify the relevance of participants to our target population. To assess respondents’ attentiveness, we incorporated attention-check items into the questionnaire (e.g., “Please select 4 = Neutral”). To minimize common method bias, data were collected in two separate waves. Participants could earn cash incentives by completing tasks at each stage, with a total reward of 6 RMB (approximately $1 USD) for full participation. In the first stage, we distributed a preliminary survey, including screening questions and questions on digital-AI transformation, to 400 respondents. In the second wave, the platform’s longitudinal tracking feature is employed, which matches participants based on the mobile phone numbers they used to register during the first wave. This function is commonly used for matching in follow-up surveys. The second-wave survey mainly assesses job insecurity, job crafting, and AI knowledge. A total of 385 participants are successfully matched across the two waves. After screening, we obtained a final sample of 370 valid questionnaires. The survey participants were predominantly female (Female = 193, SD = 0.5), with an average age in the mid-range (mean = 35.47, SD = 0.87). Educational background showed that bachelor’s and Junior college’s degree holders comprised 70% of the total sample. Industry distribution among participants indicated that the primary sectors included finance (27%), hospitality (17%), retail (15.4%), dining (14.1%), services (11.9%), transportation (8.9%), and other industries (5.7%).

### Measures

In this study, we utilized existing scales with appropriate modifications. All scales were measured on a 7-point Likert scale (1 = strongly disagree, 7 = strongly agree). Except for the digital-AI transformation scale, all scales underwent a standard back-translation process, as their original versions were in English.

The digital-AI transformation scale was adapted from Chi et al. (2020), who developed a digital transformation scale suited for the Chinese context, this scale includes three items. Job insecurity was measured using a five-item scale from Mauno et al. (2001). Job crafting was assessed with a four-item scale by Leana et al. (2009). The AI knowledge scale was based on an adaptation by He et al. (2024) of a scale from Chiu et al. (2021) and included five items. The original version of this scale was developed by Flynn and Goldsmith (1999) to measure individuals’ perceived level of subjective knowledge regarding a specific product or technology. Chiu et al. (2021) adapted it for application in the context of artificial intelligence. Subsequently, He et al. (2024) further revised the scale by retaining its five-item structure and introducing three reverse-coded items to control for response bias. The specific contents are shown in Table 1.

**Table 1:** Measurement tools and reliability.

Variable	Items	Source	Cronbach’s alpha
Digital-AI transformation	1. The company is operating business processes based on AI technologies as part of its digital transformation.	Chi et al. (2020)	0.833
	2. The company is integrating AI technologies into its digital transformation to reshape business processes.		
	3. The company’s business operations are being transformed due to digital-AI transformation.		
Job insecurity	1. I am worried about the possibility of being fired.	Mauno et al. (2001)	0.897
	2. My job is insecure.		
	3. My job is likely to change in the future.		
	4. My job is not permanent.		
	5. The thought of getting fired really scares me.		
Job crafting	1. I will Introduce new approaches to improve my work.	Leana et al. (2009)	0.762
	2. I will Change minor work procedures that i think are not productive.		
	3. I will Change the way i do my job to make it easier to myself.		
	4. I will Rearrange equipment and change my working environment.		
AI knowledge	1. I know pretty much about AI.	Chiu et al. (2021) He et al. (2024)	0.904
	2. I do not feel very knowledgeable about AI (Reverse coded).		
	3. Among my circle of friends, I’m one of the “experts” on AI.		
	4. Compared to most other people, I know less about AI (Reverse coded).		
	5. When it comes to AI, I really do not know a lot (Reverse coded).		

## Results

### Confirmatory factor analyses

Using AMOS 24.0 software, confirmatory factor analysis (CFA) was conducted to evaluate the four variable dimensions. The results show that, the four-factor model demonstrated satisfactory fit indices (χ2/df = 2.878 < 3, CFI = 0.945 > 0.9, TLI = 0.934 > 0.9, GFI = 0.904 > 0.9, NFI = 0.919 > 0.9, RMSEA = 0.071 < 0.08, SRMR = 0.061 < 0.08), indicating significantly better fit compared to other factor models. These results suggest that the variables in the model possess good discriminant validity.

### Common method variance

Given that the theoretical model in this study is based on self-reported data, Harman’s single-factor test was conducted using SPSS 22.0 to control for common method bias. Results showed that the first factor explained 25.591% of the variance, which is below the recommended threshold of 40%, indicating that common method bias is not a significant concern (Podsakoff et al., 2003).

### Descriptive statistical analysis

Table 2 shows the mean, standard deviations, and correlation coefficients of each variable.

**Table 2:** Descriptive statistics and correlations.

Variables	M	SD	Sex	Age	Education	DAT	JI	AK	JC
Sex	1.52	0.50	1						
Age	3.00	0.87	0.001	1					
Edu	3.79	0.89	0.031	0.083	1				
DAT	3.99	1.59	−0.005	−0.027	0.100	1			
JI	4.54	1.47	−0.013	−0.079	−0.046	0.577**	1		
AK	4.31	1.57	0.001	−0.009	0.115*	0.360**	0.335**	1	
JC	4.23	1.28	0.003	−0.051	0.032	0.632**	0.686**	0.372**	1

*N* = 370, DAT, digital-AI transformation, JI, job insecurity, JC, job crafting, AK, AI knowledge, *p < 0.05; **p < 0.01.

### Hypotheses testing

Using SPSS PROCESS 3.3 Model 4, we tested the total effect of digital-AI transformation on job crafting as well as the mediating effect of job insecurity. The results, shown in Table 3, indicate a significant total effect of digital-AI transformation on job crafting [Effect = 0.512, 95% CI = (0.447, 0.576)], supporting Hypothesis 1. Additionally, job insecurity significantly mediates the relationship between digital-AI transformation and job crafting [Effect = 0.228, 95% CI = (0.176, 0.288)], confirming Hypothesis 2.

**Table 3:** Results of mediation analysis.

Path	Effect	SE	95% LLCI	95% ULCI
Total effect				
DAT → JC	0.512	0.033	0.447	0.576
Direct effect				
DAT → JC	0.283	0.035	0.214	0.352
Indirect effect				
DAT → JI → JC	0.228	0.029	0.176	0.288

*N* = 370, DAT, digital-AI transformation; JI, job insecurity; JC, job crafting.

**Figure 2:**
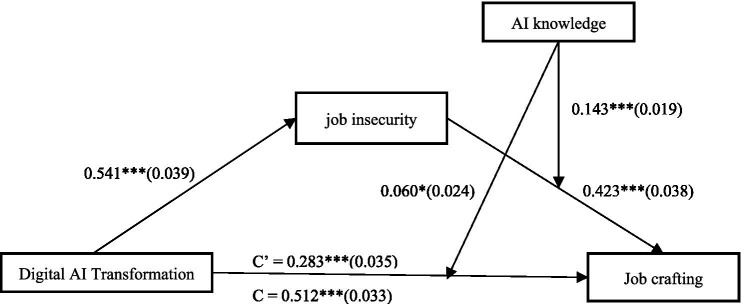
Structural model. ***p < 0.001, *p < 0.05. C′ is the indirect effect, C is the total effect.

Using SPSS PROCESS 3.3 Model 15, we tested the moderating effect and moderated mediation effect of AI knowledge. The results shown in Table 4 indicate that AI knowledge has a significant positive effect on job crafting (*β* = 0.170, *p* < 0.001). Additionally, a significant positive interaction between digital AI transformation and job insecurity was observed in T2 (β = 0.060, *p* < 0.05), supporting Hypothesis 3 (Figure 2).

**Table 4:** Results of path analysis.

Variable	T1	T2	T3	T4
	JC	JC	JI	JC
	*β* (se)	*β* (se)	*β* (se)	*β* (se)
Sex	0.007 (0.102)	0.021 (0.101)	−0.021 (0.124)	0.0422 (0.083)
Age	−0.031 (0.058)	−0.046 (0.058)	−0.092 (0.071)	−0.006 (0.048)
Edu	−0.043 (0.058)	−0.062 (0.057)	−0.163 (0.070)	−0.006 (0.047)
DAT	0.574*** (0.034)	0.449*** (0.035)	0.541*** (0.039)	0.190*** (0.034)
JI	\	\	\	0.444***(0.036)
AK	0.170*** (0.036)	0.167***(0.036)	\	0.150***(0.030)
DAT*AK	\	0.060*(0.024)	\	\
JI*AK	\	\	\	0.143***(0.019)
*R* ^2^	0.418	0.436	0.346	0.623
*F*	54.058***	46.848***	48.338***	85.263***

*N* = 370, DAT, digital-AI transformation; JI, job insecurity; JC, job crafting; AK, AI knowledge, ***p < 0.001, **p < 0.01, *p < 0.05.

**Figure 3:**
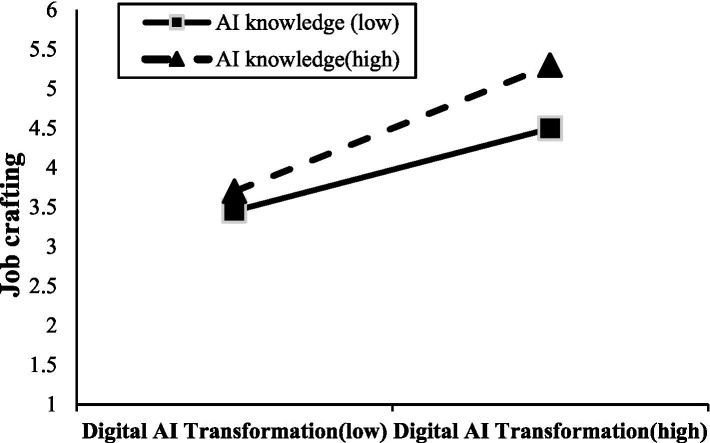
The moderating effect of AI knowledge.

To further investigate the moderating effect of AI knowledge, a simple slope analysis was conducted. As shown in Figure 3 and Table 5, when AI knowledge is at a low level (mean − SD), digital-AI transformation has a significant positive impact on job crafting [*β* = 0.354, 95% CI = (0.247, 0.461)]. When AI knowledge is at a high level (mean + SD), digital-AI transformation also has a significant positive impact on job crafting [*β* = 0.544, 95% CI = (0.453, 0.653)]. This indicates that as the level of AI knowledge increases, the impact of digital-AI transformation on job crafting also strengthens.

**Table 5:** The moderating effect and moderated mediating effect of AI knowledge.

Adjustment path	AK	*β*	Boot SE	Boot LLCI	Boot ULCI
DAT → JC	Mean − 1SD	0.354	0.055	0.247	0.461
	mean	0.449	0.035	0.381	0.517
	Mean +1SD	0.544	0.046	0.453	0.635
DAT → JI → JC	Mean –1SD	0.118	0.031	0.060	0.182
	mean	0.240	0.027	0.190	0.296
	Mean + 1SD	0.362	0.035	0.296	0.434
	Index	0.078	0.012	0.055	0.103

*n* = 370, DAT, digital-AI transformation; JI, job insecurity; JC, job crafting; AK, AI knowledge.

As shown in Table 4, T3 reveals a significant positive effect of digital-AI transformation on job insecurity (*β* = 0.541, *p* < 0.001), and Model 4 shows a significant positive interaction effect of job insecurity and AI knowledge on job crafting (*β* = 0.143, *p* < 0.001). Thus, combined with the support for Hypothesis 2, these results indicate that AI knowledge moderates the mediating effect of job insecurity between digital-AI transformation and job crafting, confirming Hypothesis 4 (Figure 2).

**Figure 4:**
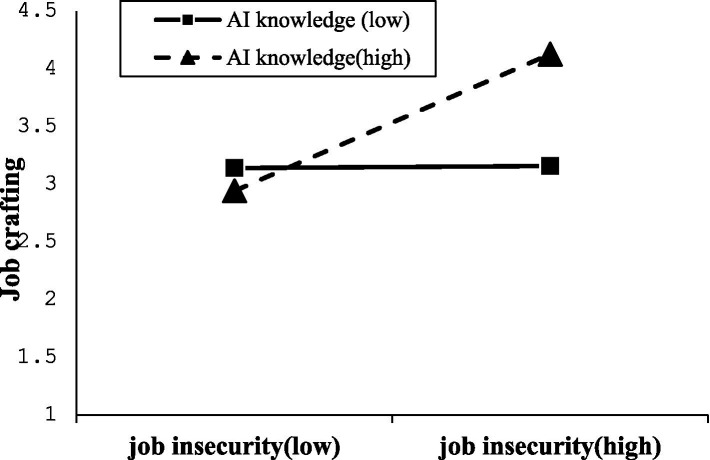
The moderated mediating effect of AI knowledge.

To further examine the moderated mediation effect of AI knowledge, a simple slope analysis was conducted, as illustrated in Figure 4 and Table 5. When AI knowledge is at a low level (mean - SD), job insecurity has a significant positive mediating effect between digital-AI transformation and job crafting [*β* = 0.118, 95% CI = (0.060, 0.182)]. When AI knowledge is at a high level (mean + SD), job insecurity also has a significant positive mediating effect between digital-AI transformation and job crafting [*β* = 0.362, 95% CI = (0.296, 0.434)]. This indicates that as AI knowledge increases, the mediating effect of job insecurity in the relationship between digital-AI transformation and job crafting also strengthens.

The results of the hypothesis verification of this study are shown in the Table 6.

**Table 6:** Study hypothesis testing results.

Hypothesis	Paths	Path coefficients	Results
H1	DAT → JC	0.512	Supported
H2	DAT → JI → JC	0.228	Supported
H3	DAT*AK → JC	0.060	Supported
H4	JI*AK → JC	0.143	Supported

DAT, digital-AI transformation; JI, job insecurity; JC, job crafting; AK, AI knowledge.

## Discussion

With the continuous development of AI technology, AI-driven digital transformation has become a new direction, often accompanied by a range of human resource management challenges. Framed by Conservation of Resources Theory, this study investigates how digital-AI transformation impacts employees’ job crafting behaviors and explores the underlying drivers of this behavior. The findings of this study contribute meaningfully to the Conservation of Resources Theory by extending its application to the context of digital-AI transformation—an emerging organizational setting. First, the study highlights that perceived resource threats, under certain conditions, can activate adaptive behaviors such as job crafting. Second, AI knowledge is conceptualized as a critical personal resource in this context, functioning both as a buffer against resource loss and as a resource amplifier. Moreover, drawing on Conservation of Resources Theory, this study emphasizes that organizations should leverage AI knowledge to stimulate employees’ job crafting, rather than relying on job insecurity as a motivator, in order to avoid potential ethical violations.

### Theoretical implications

Firstly, this study offers a new perspective on the impact of AI-driven digital transformation within the field of human resource management. With the rapid advancement of AI technology, many organizations are now integrating AI applications as a core component of their digital transformation strategies (Liang et al., 2022; Yang et al., 2024). However, the environmental volatility associated with technological change has a substantial impact on employees’ psychological states and behaviors. While existing literature has examined some employee responses to organizational AI adoption—such as AI anxiety and AI awareness (Li et al., 2019; Wang and Wang, 2022)—research into the effects of organizational AI adoption on employees remains limited. Therefore, drawing on Conservation of Resources Theory, this study examines employees’ psychological and behavioral responses during digital-AI transformation, addressing a key gap in the literature.

Secondly, our findings reveal that digital-AI transformation has a significant positive impact on job crafting. Previous research has noted that, while the introduction of AI may induce technological anxiety among employees, this anxiety can also motivate job crafting efforts (Tan et al., 2024). For example, Wang et al. (2025) also confirm that employees in AI-transformed organizations tend to engage in job crafting behaviors. Moreover, such technological change does not appear to be significantly influenced by national context (He et al., 2023). Conversely, some studies suggest that AI-induced anxiety may have adverse effects on employees (He et al., 2024). This may be attributed to the organizational context in which employees are situated, such as those in the service industry (He et al., 2024; Li et al., 2019; Sha et al., 2025). Thus, our research aligns partially with existing studies, confirming the double-edged nature of AI technology’s impact (Liang et al., 2022). We interpret the positive impacts of digital-AI transformation through the conservation of resources framework. Digital-AI transformation can be perceived by employees as either a threat or an opportunity. When employees sense a potential resource threat from organizational technological changes, they tend to adapt their behaviors to better align with organizational shifts. Alternatively, they may view this transformation as an opportunity, encouraging them to modify their work approaches more proactively. This perception aligns with prior digital transformation experiences, as digital-AI transformation builds upon digital initiatives by incorporating AI technology (Fenwick et al., 2024). Employees with prior digital transformation experience are more likely to view digital-AI transformation as an opportunity to gain new resources. Our research enhances understanding of the antecedents of job crafting in digital transformation contexts and suggests that organizations in digital transformation actively incorporate AI technology.

Third, while previous research on the impact of AI has largely focused on its negative effects on employees (Wang and Wang, 2022; Wang et al., 2022), this study explores the positive impacts of AI by introducing job insecurity as a variable to examine employees’ motivations for job crafting within the context of AI-driven digital transformation. Prior studies suggest that AI can provoke job insecurity and potentially reduce service performance (He et al., 2024), which differs from our findings. Although digital-AI transformation may induce feelings of threat and job insecurity among employees, these emotions do not always lead to negative outcomes. The inconsistency in results may be attributed to industry differences among organizations implementing AI. Previous studies often focus on sectors with high job substitutability, such as the service industry (Chen and Cai, 2025). Additionally, the specific context of digital-AI transformation in the current study may contribute to the observed effects. Employees who have experienced digital transformation are more likely to perceive AI-driven changes as opportunities rather than threats. Wang et al. (2025) also emphasize that members of digitally transformed organizations tend to interpret AI-related challenges as opportunities, thereby engaging in job crafting behaviors. According to Conservation of Resources Theory, when individuals face resource threats, their stress perception systems intensify (Bickerton and Miner, 2023), manifesting as job insecurity. This sense of stress may, in turn, activate employees’ motivation to protect resources, prompting them to consciously adapt their work practices. Our findings indicate that job insecurity serves a positive mediating role between digital-AI transformation and job crafting. Nevertheless, a large body of research emphasizes the detrimental consequences of job insecurity, such as reduced service performance, diminished sense of responsibility, and increased anxiety (He et al., 2024; Sun et al., 2022; Wu et al., 2024). These studies highlight that job insecurity is typically regarded as a harmful psychological state. Therefore, the potential positive effects of job insecurity should be critically reconsidered. Although job insecurity may temporarily motivate employees to engage in job crafting, it should not be viewed as a desirable form of motivation. Prolonged exposure to job insecurity not only fails to sustain beneficial outcomes but also exacerbates psychological distress, ultimately resulting in serious organizational consequences (Reisel et al., 2010). Thus, organizations should not consider or employ job insecurity as a legitimate motivational tool.

Fourth, grounded in the Conservation of Resources Theory, this study highlights the importance of AI knowledge as a personal resource in the process of digital-AI transformation and expands the understanding of AI knowledge. This finding helps prevent organizations from adopting job insecurity as a strategic tool, thereby reducing the risk of violating ethical standards. AI knowledge enhances employees’ positive perceptions of AI within digital transformation, motivating them toward proactive job crafting (He et al., 2024). Additionally, as a form of personal resource, AI knowledge can be replenished even amidst resource depletion. Our findings confirm the positive moderating role of AI knowledge in the relationship between digital-AI transformation and job crafting. AI knowledge also moderates the mediating role of job insecurity between digital-AI transformation and job crafting, an effect that relates to the limitations of AI technology. Employees with higher AI knowledge better understand AI’s current capabilities, recognizing that AI cannot entirely replace human roles in the near term (Abdullah and Fakieh, 2020; He et al., 2024; Oh et al., 2019). Thus, AI knowledge helps mitigate the negative impact of job insecurity, even transforming it into a driver of positive behaviors. This study broadens the conceptualization of AI knowledge and suggests that organizations emphasize AI knowledge development among employees during digital-AI transformation.

### Practical implications

This study offered several managerial implications for the field of AI-driven digital-AI transformation. First, the findings indicated that employees could respond proactively to digital-AI transformation by engaging in job crafting. Therefore, organizations currently undergoing digital transformation might consider actively integrating AI technologies. Not only could AI enhance operational efficiency, but it also constituted a crucial component of future strategic development. Notably, while AI provided significant advantages in addressing organizational complexity and uncertainty, its integration should be approached cautiously (Jarrahi, 2018), given the double-edged nature of AI’s effects (Dong et al., 2024).

Secondly, this study revealed that job insecurity plays a positive mediating role between digital-AI transformation and job crafting. Although job insecurity is typically viewed as harmful, the findings of this study confirm that, under certain contextual conditions, it may serve as a catalyst for job crafting. However, it should not be regarded as a strategic tool. Numerous studies have demonstrated that prolonged job insecurity undermines employee well-being and ultimately weakens organizational performance (Mauno et al., 2001). Job insecurity often emerges as an inevitable emotional response during digital-AI transformation. Given its dual nature, organizations are advised to foster transparent communication, provide AI-related training, and establish support systems to maintain a psychologically safe environment, ensuring that employees’ stress levels remain within a manageable range.

Thirdly, the research findings underscore the moderating role of AI knowledge in digital-AI transformation and its regulatory effect in the mediating process. This indicates that organizations should actively promote AI knowledge prior to implementing digital-AI transformation. For example, organizations can enhance AI skills training and offer online AI courses, which can help employees reevaluate their understanding of AI and maintain clarity during technological changes. Additionally, these initiatives assist employees in grasping the actual state of AI technology development, thereby mitigating negative impacts stemming from exaggerated perceptions of AI (Jarrahi, 2018). Moreover, organizations are encouraged to recognize the strategic importance of AI knowledge within digital-AI transformation initiatives. AI-related training should be systematically integrated into organizational learning and development programs to continuously enhance employees’ AI knowledge. This approach not only reduces resistance to digital-AI transformation but also strengthens employees’ adaptability and proactivity, thereby facilitating a smoother transition toward a digital-AI-driven operational model.

### Limitations and future research

This study has certain limitations. First, data collection was restricted to a single country, which may limit the generalizability of the findings. Future research could consider cross-national samples to enhance the applicability of the results. Second, there may be bias in the job insecurity variable, as our sample includes employees from various levels, including senior management. Due to their higher organizational status, senior managers may not perceive threats associated with digital-AI transformation (Reisel et al., 2010). Third, although a two-wave survey design is adopted, the data primarily rely on self-reported measures, which may introduce common method bias. Future studies are encouraged to incorporate mixed-method longitudinal designs, such as behavioral observations or interviews, to reduce the potential bias associated with self-reporting. Finally, the findings suggest that digital-AI transformation exerts a dual effect on employees, which may be closely related to industry-specific contexts. Therefore, future research should conduct comparative studies across industries undergoing AI-driven change to deepen the understanding of its organizational impact.

## Conclusion

This study empirically examines the relationship between digital-AI transformation and job crafting, as well as its underlying mechanisms, through the lens of the Conservation of Resources Theory. Our findings reveal that digital-AI transformation stimulates employees’ job crafting both directly and indirectly through job insecurity. Additionally, AI knowledge serves as a personal resource that moderates these relationships. These insights offer a deeper understanding of employee behavior during digital-AI transformation and provide practical management implications for organizations integrating AI within their digital transformation processes.

## Data Availability

The raw data supporting the conclusions of this article will be made available by the authors, without undue reservation.
